# College Commencements and Alumni Association Meetings

**Published:** 1894-06

**Authors:** 


BUFFALO MEDICAL AND SURGICAL JOURNAL

A MONTHLY REVIEW OF MEDICINE AND SURGERY.

*EDITORS:*

THOMAS LOTHROP, M. D. - - WM. WARREN POTTER, M. D.

All communications, whether of a literary or business nature, should be addressed to the managing editor: 284 FRANKLIN STREET, BUFFALO, N. Y.

VOL. XXXIII. JUNE, 1894. No. 11.

### COLLEGE COMMENCEMENTS AND ALUMNI ASSOCIATION MEETINGS.

### BUFFALO UNIVERSITY MEDICAL COLLEGE.

The exercises connected with the annual commencement of this institution fell this year on May 1st, and it was in every way a fit day and a pleasant occasion. For forty-eight years these ceremonies have recurred, and with increasing interest to our citizens as well as the college authorities and pupils.

The Alumni Association began its nineteenth annual session at 11 o'clock A. M., with the president, Dr. W. C. Phelps, of Buffalo, in the chair. The roster was read, and those in attendance responded, after which the minutes of the last annual meeting were read by the recording secretary, Dr. F. B. Willard, of Buffalo.

The committee on necrology reported the following deaths: Dr. William F. Hutchinson, of the class of '73, at Providence, R. I., on September 30, 1893; Dr. Warner C. Bush, Branchport, N. Y., September 29, 1892; Dr. Ganson W. Croff, class '67, at Bethany, N. Y., March 21, 1893; Dr. D. C. Case, class '65, at Howard, N. Y., April 4, 1894; Dr. Charles L. Dayton, class '53, in Buffalo, September 7, 1893; Dr. Charles W. Davis, class '87, at Chautauqua, N. Y., April 9, 1894; Dr. William B. Gould, class '48, at Lockport, June 20, 1893.

Dr. A. L. Benedict suggested an amalgamation of the several alumni associations of the University for the purposes of social entertainment and acquaintance. After a discussion of the subject, a motion prevailed to appoint a committee to confer with the other organizations, and the president appointed Drs. Rochester, Jones and Parmenter as such committee.

In behalf of the committee appointed to secure subscriptions to the college fund, Dr. Rochester, in the absence of Dr. Putnam, reported that $2,200 had been subscribed, and of this amount $1,820.85 was available. The committee was discharged and a resolution passed ordering the list of subscriptions and the moneys paid in turned over to the officials of the University. The report of the treasurer showed a balance on hand of $28.51 and that $59 had been expended during the year.

The following officers were elected: President, Dr. Ernest Wende, Buffalo; first vice-president, Dr. William Baker, Warren, Pa.; second vice-president, Dr. P. W. Van Peyma, Buffalo; third vice-president, Dr. Jane W. Carroll, Buffalo; fourth vice-president, Dr. D. A. Currie, Englewood, N. Y.; fifth vice-president, Dr. McPherson, Tonawanda;—permanent secretary, Dr. E. L. Frost, Buffalo;—recording secretary, Dr. N. Victoria Chapell;—treasurer, Dr. H. U. Williams;—executive committee, Dr. H. H. Bingham, chairman;—Dr. A. T. Lytle, secretary; Dr. F. B. Willard;—trustee, Dr. Julius Wenz, Lancaster.

At the afternoon session an address of welcome was delivered by Dr. M. D. Mann, Dean, which was responded to by the president. The following program was then carried out: The Etiology, Pathology and Prognosis of Certain Phases of Gonorrhea,—Dr. George Emerson Brewer, assistant demonstrator of anatomy, College of Physicians and Surgeons, New York, member American Association of Genito-urinary Surgeons. Pancreatitis,—Charles G. Stockton, M. D., professor principles and practice of medicine, University of Buffalo. The Treatment of Several Common Ailments and Particularly the Relationship between Croupous Pneumonia as an Infectious Process and its Therapeutics,—H. A. Hare, M. D., professor of therapeutics in the Jefferson Medical College, Philadelphia. The Medical Examination of the Living Human Body when required by Courts,—Tracy C. Becker, Esq., professor medical jurisprudence, law department, University of Buffalo; president New York State Bar Association and legal editor of a standard Treatise on Medical Jurisprudence, Forensic Medicine and Toxicology.

The commencement exercises were held in Music Hall. After an orchestral selection the exercises were opened by the presentation of the candidates for the degree of Doctor of Medicine to the vice-chancellor, Mr. George S. Hazard, who officiated in the place of the chancellor, the Hon. E. C. Sprague, who was unable to be present owing to illness. Dr. M. D. Mann administered the Hippocratic oath, which obligated the class to the strict fulfillment of their duties as medical practitioners, after which the members of the class marched upon the stage and received their diplomas from the hands of Mr. Hazard. The graduates in medicine were the following: Nelson O. Brooks, Frank T. Carmer, Eugene T. Caswell, John Chalmers, Timothy T. Clohessy, La Verne C. Colegrove, Myron E. Fisher, Jane North Frear, William Gaertner, Ph. D., Benjamin A. Gipple, Frederick B. Green, Edward R. Hardenbrook, M. D., A. E. Hubbard, J. Grafton Jones, Henry E. Long, Cyrus P. Jennings, A. W. Jackson, M. D., Cora Billings Latten, M. P. Messenger, John L. Miller, Harrison C. Potter, Herriot C. Rooth, Ernest L. Ruffner, George W. Sales, Angeline D. Smith, Amelia Earl Trant, Charles F. Tucker, Frederick J. Tunmore, Lewis A. Twining.

The announcement of the honors then followed, and as the names of Messrs. Myron E. Fisher, William Gaertner, Ph. D., Frederick B. Green, J. Grafton Jones, Cyrus P. Jennings, M. P. Messenger, Herriot C. Rooth, Ernest L. Ruffner, Amelia Earl Trant, Cora Billings Latten and Jane North Frear were called, the audience greeted them with applause, which was especially hearty when the names of the women were announced.

The department of pharmacy granted diplomas to twenty-six, and the department of dentistry to six students.

Dr. Lucien Howe, professor of ophthalmology, then addressed the graduated classes. In the course of his remarks he quoted an editorial comment from the *London Spectator*, which, speaking for England, said that of all who start on professional careers one-third go under, that is, get sick, die or emigrate; one-third barely survive, fighting on without a hope of retiring in old age, and one-third make a decent and comfortable living. Sir James Paget, who had made a study of the subject and had compiled some important statistics, had said that of 1,000 medical students whose careers had been investigated, 23 had obtained eminence, 66 had considerable success, 507 were fairly successful, 124 had exceedingly limited practice, 56 failed entirely, 96 or nearly one-tenth of the number abandoned the profession and 87 died after entering upon the practice of medicine, and 5 of these had committed suicide.

In conclusion, he spoke of the great debt of gratitude the world owed the medical profession for its discoveries and its scientific advancement by means of which human life is prolonged. The average length of life is now forty years. Seventy years ago it was thirty years, and this extension of ten years, or one quarter of the average life, is due to the persistent investigations of men who worked in their profession for the love of it. His final words to the students were an admonition to them to go forth with the determination to work, and by that means become a credit to their Alma Mater, as well as achieving a grand and glorious success.

The annual alumni banquet was given at the Iroquois under the toastmastership of Dr. F. P. Vandenbergh, whose function was discharged with grace and wit. The following is the list of subjects and speakers: The Class of '94, John Chalmers, M. D.; The University of Buffalo, Dr. Roswell Park.; The Environs of Buffalo, the Hon. Cuthbert W. Pound; Tact and Opportunity in Professional Life, Mr. Edward C. Randall; Leisure Thoughts of a Busy Man, Mr. Frank E. Blackwell; Side-lights on Some Topics, Mr. Samuel G. Blythe; The Growth and Prospects of Buffalo, Mr. William C. Cornwell.

Several women physicians lent the grace of their presence at the banquet, which was altogether a most pleasant and inspiriting addition to the festive scene.

### NIAGARA UNIVERSITY MEDICAL COLLEGE.

The commencement exercises of this institution, like those of its elder sister, began with the annual meeting of the alumni association. The morning session was called to order at 10.30 o'clock, May 9, 1894, in the amphitheater of the medical college, by the president, Dr. John M. Hewitt, of Buffalo, who delivered an address upon medical jurisprudence immediately after Dr. Cronyn's address of welcome.

Then followed a business session, at which the following named were elected officers for the ensuing year: President, Dr. E. M. Dooley, Buffalo; first vice-president, Dr. J. F. Meyer; second vice-president, Dr. F. A. Hayes; secretary, Dr. W. H. Norish; treasurer, Dr. E. E. Martin; executive committee, Dr. J. H. Dowd, Dr. C. J. Reynolds and Dr. S. A. Dunham.

During the afternoon session that convened at 3 o'clock, the following program was carried out:

1. Maritime Quarantine, by William A. Wheeler, M. D., Surgeon U. S. M. H. S., formerly professor of surgery Niagara University. (See p. 659, this journal.)

2. Rhinoplastic Surgery, by Daniel F. Sullivan, M. D., Hartford, Conn.

3. Some Experiences, by Howard L. Hunt, M. D., Orchard Park, N. Y.

The exercises incident to the conferring of degrees were held at the Academy of Music, commencing at 8 o'clock P. M. On the stage were seated the Rt. Rev. Stephen V. Ryan, D. D., the Rev. Henry Elliott Mott, pastor of the Central Presbyterian Church, members of the faculty of the medical department and of the university and guests from out of town.

The ceremonies began with a valedictory address by Harlow C. Curtiss, Esq., professor of medical jurisprudence. [To be published.] Upon the conclusion of Prof. Curtiss's address, Dr. John Cronyn administered the usual pledge, differing in form from the Hippocratic oath, to the graduates. This was followed by the conferring of degrees by the Chancellor, the Rt. Rev. Bishop Ryan, by hooding the candidates, an ancient rite which is practised in many foreign universities.

The graduates to receive diplomas this year were George Edward Albon, Robert Jacob Baxter, Buffalo; Francis Joseph Carr, Greenwood; Jasper Glenn Ernest, Lockport; Fred. S. Hoffman, Johnson's Creek; Max Kaiser, Edward Edwin Koehler, Earl Perkins Lothrop, Daniel Vincent McClure, Buffalo; Francis William McGuire, Mount Forest, Ont.; James William Nash, Henry Osthues, Buffalo; Jeremiah Henry Walsh, Curtiss; Henry James Williams, Buffalo.

The graduates upon whom were bestowed special honors this year were Earl Perkins Lothrop, Max Kaiser and Fred S. Hoffman. Among the graduates in whom more than ordinary interest was felt by his friends and those who understood the obstacles that he has had to overcome, was Henry James Wilson, who has pursued his medical studies while employed by the Government as one of the mounted night collectors of mail for the Postoffice.

After the bestowal of degrees, the address to the graduates was delivered by the Rev. Henry Elliott Mott who quickly aroused the interest and enthusiasm of the audience by saying that physicians are a handy thing to have around once in a while and that he hoped to clinch his hold on them in the unfolding of his theme, which he should entitle, Damon and Pythias, or the Physician and the Parson. He had meant, he said, to introduce a third functionary in the person of an undertaker, but had concluded to leave him out. Taking the physician and the parson as his theme, the speaker delivered an address of absorbing interest, in which frequent flashes of wit illumined his words of wisdom. His plea was for optimism and a perfect trust in God. The soul, he said, is as sensitive to influences as are the muscles, and he urged the graduates before him not to give away to materialistic philosophy. The exercises were concluded with a few words from the chancellor, the Rt. Rev. Bishop Ryan.

Then followed the annual banquet at the Tifft House, where the faculty, graduates and invited guests, to the number of about eighty, sat down to tables laden with good things and handsomely decorated. There were present, as guests of the Association, the members of the Board of Medical Examiners, including Dr. F. W. Ross, of Elmira; Dr. Morris N. Bemis, of Jamestown, and Dr. Edward Torrey, of Allegany. Among the toasts responded to were: Niagara University, by the Rev. L. A. Grace; The Press, by F. W. Kendall; The War of the Experts, by Dr. John A. Miller; The Medical Profession, by Dr. Byron H. Daggett; The Bald-headed Man in Physics, by Dr. Carlton C. Frederick; The Graduating Class, by Dr. Max Kaiser.

Dr. Daggett's response to the medical profession, teeming with wit and brilliancy, is worthy of preservation, and we herewith reproduce it in full:

An invitation to respond at the banquet of the Alumni Association of the University of Niagara was sufficiently flattering to overcome my prudence, and I promptly accepted. A little reflection caused me to repent of my rash promise to attempt to say something new, useful or interesting upon such a subject and to such an audience. The theme is so comprehensive that its contemplation appalled me, and I resolved to follow the example of Nasby who was announced to lecture on the Babes in the Wood, and never said a word about the babes.

Having disposed of the subject, the audience still confronts me, and the only apparent means of escape was a request to the toastmaster not to call upon me until you had reached such a degree of hilarity that you would kindly receive stumbling rhetoric and stale humor.

It is stated that medicine is coëval with man, which reminds me that once upon a time I read of the escapade in the garden, which brought disaster upon our kind, sent our progenitors house-hunting, and made them so ashamed that they covered themselves with a fig leaf. This established a precedent for Anthony Comstock.

In due time after the installation of the lares and pennates of this first family in their new home, the good husband and prospective father, during the still hours of the night, was nudged in the floating ribs and told to telephone the doctor. There is no evidence that there were skilled obstetricians at that time, as the Medical Department of the University of Niagara had not yet been instituted.

The kind husband evidently administered during this laborious affair, ligated and severed the cord of life, and dropped the "tiny feather from the wing of love into the sacred lap of motherhood." Thus the ancient origin of obstetrical art is established and given most honorable precedent. As this little ootsy-tootsy waxed and grew strong, he, like the modern article, got wind on his stomach, causing tormina and midnight uproar, which were soothed by catnip tea and anise, and we have the beginning of pediatrics.

The record of the profession during the following antediluvian ages is shrouded in the mists of antiquity. Medicine had its birthright in the ancient continent, that breeder of epidemics, that land of dead civilizations and living barbarisms, that mother of religions and home of idolators, whose 800,000,000 of tawny inhabitants vegetate in a pall of mental torpor, whose greatness is moldering in musty sepulture, or towers in broken columns and crumbling capitals in desert wastes,—stony epics of the olden time!—dread harbingers of fate, giving out Memnonian oracles from the else forgotten past.

We find in the débris of these dead civilizations, and in the relics of prehistoric races, abundant evidence of the beneficent activity of the doctor. Lithotomy is older than Hippocrates; mercury, sulphur and stramonium were known long before the days of Esculapius and Galen; long before hundred-gated Thebes had blessed the world with her cult, or Nineveh had donned her swaddling clothes.

The late doctor, Madam Semiramis, Queen of Assyria, was the inventor of orchidectomy and mother of andrology. Andrology has lagged in the procession of the specialties, gynecology is winning the honors and emoluments, but the female physician is abroad in the land, and should she specialize andrology she would find hosts of weeping tubes which bode suppurating woe in Fallopian ways; anticipate and forestall oöphorectomy by doing orchidectomy as a sanitary measure. Posthetomy is as old as the Jews and was common in Egyptian civilization.

Ricord said that in the beginning the heavens, the earth, man and venereal diseases were created, and ancient history shows that devotees of Venus early sought penance at the throne of Mercury. Moses as a sanitary measure commanded the slaughter of 24,000 people who had worshiped at the shrine of Baal-Peor. The modern Moses is less sanguinary in his methods; he leads not the infected to slaughter by the sword; he pickles the infection by baptism in bromin.

We read in sacred history of the good doctor Luke, whose praise is in the gospel of all the churches; also of Asa, who became sorely afflicted in the feet. He sought not the Lord, but the physicians, and Asa slept with his fathers.

> A wise physician, skilled our wounds to heal;
> Is more than armies to the public weal—

sang the poet of the Trojan war.

During the late Civil War, our victorious armies marched safely through the malarious valleys of the South attended by the skilled Yankee doctor, who was indeed "more than armies to the public weal." Paul F. Eve, medical director of the Confederate Army, stated that the want of quinine contributed powerfully to the overthrow of the Confederate forces, and that he tried, in vain, sixty substitutes for this remedy. Ben Butler and his medical staff made New Orleans a respectful and healthful city in spite of the rebel confession and boast that yellow jack was mightier than Confederate hosts.

Westward the course of medicine, like empire, takes its way, and its grandest triumphs have risen in Western civilization. William Pepper asserted at the Pan-American Medical Congress that medicine had advanced more in the past twenty years than in the previous 2,000.

Unfortunately modern medicine is hampered by "isms" and "pathies." The so-called allopathic or regular system is truly eclectic and accepts any rational remedy, whether derived from the earth, the air, or the waters under the earth,—a platform broad enough for all.

> The homeopathic system, sir, just suits me to a tittle,
> Anyhow, it proves of physic you cannot take too little;
> If it be good in all complaints to take a dose so small,
> It must be better still to take no dose at all.

The following gems are taken from the *American Homeopathist* for April, 1893:—

If patient has head always turned to one side, cina is indicated.

If patient sleeps with knees apart, chamomilla.

If patient sleeps with legs stretched out at full length, pulsatilla and rhus are indicated.

If patient bends the head forward, staphisagara; if backward, hyoscyamus.

If patients lie with hands on the abdomen, pulsatilla.

If patient sleeps with one leg drawn up and the other stretched out, stannum is indicated. (I am not up enough in therapeutics to know whether tin draws up stretched-out legs, or stretches out drawn-up legs.)

If a woman sleeps with her hands over her head, pulsatilla.

If a man sleeps with his hands over his head, nux.

If a patient has a cold red cheek and a pale hot cheek, moscus.

If a patient gets suddenly very much better, it is a bad sign.

If a patient "cusses you," spits in your face, and pulls your whiskers, chamomilla.

We have also the faith cure and the mind cure, which were aptly differentiated by Dudley Warner, when he said: "The faith cure requires no mind, and the mind cure requires no faith."

Hydropathy is a valuable annex, which received a fitting tribute from the dying Dumoulin, who said, "I leave behind me three excellent physicians." His weeping colleagues bowed, when to their consternation he named, water, exercise and regimen. Through all the ages and through all the 'pathies these remedial agents have stood the test.

Modern medicine is preëminently preventive. The tireless energy of skilled medical endeavor is constantly laboring to solve the problems of life and of health; studying ways of rendering the system immune; developing methods of relieving existing disease; promoting longevity, so that man's days shall be 120 years, and wooing Euthanasia, in order that old age may bear neither pain, passion nor sorrow, as step by step the intéllect wanes and activity lapses into repose. "The merry sounds of youth and the hum of the busy world only rock gently to sleep as consciousness gradually ceases; death suggesting no terror, inflicting no pain, bringing no agony."

Modern medicine has formed the profession in the right of the line of that grand army of the arts and sciences which have budded and blossomed in the brilliant, electrical splendor of the Christian civilization of the nineteenth century, and which are preparing man for that perfect day, when the nations of the earth shall beat their swords into plow shares; their scalpels into pruning hooks; throw physic to the dogs, and never more know war, pestilence, pain or sorrow.

This clever speech was frequently interrupted by outbursts of hearty applause.

